# When Ribonucleases Come into Play in Pathogens: A Survey of Gram-Positive Bacteria

**DOI:** 10.1155/2012/592196

**Published:** 2012-03-13

**Authors:** Brian C. Jester, Pascale Romby, Efthimia Lioliou

**Affiliations:** Architecture et réactivité de l'ARN, UPR 9002 CNRS, IBMC, Université de Strasbourg, 15 rue René Descartes, 67084 Strasbourg, France

## Abstract

It is widely acknowledged that RNA stability plays critical roles in bacterial adaptation and survival in different environments like those encountered when bacteria infect a host. Bacterial ribonucleases acting alone or in concert with regulatory RNAs or RNA binding proteins are the mediators of the regulatory outcome on RNA stability. We will give a current update of what is known about ribonucleases in the model Gram-positive organism *Bacillus subtilis* and will describe their established roles in virulence in several Gram-positive pathogenic bacteria that are imposing major health concerns worldwide. Implications on bacterial evolution through stabilization/transfer of genetic material (phage or plasmid DNA) as a result of ribonucleases' functions will be covered. The role of ribonucleases in emergence of antibiotic resistance and new concepts in drug design will additionally be discussed.

## 1. Introduction

Bacterial pathogens predominantly respond to environmental changes, such as entry into a host, by adapting their physiology through altered gene expression. The gene products that give a pathogen an enhanced chance of survival within the host are termed virulence factors. Pathogens use a variety of different mechanisms to regulate virulence gene expression. Besides transcriptional control, several post-transcriptional mechanisms have been well documented in the literature [1, 2]. In the recent years, messenger RNA (mRNA) stability emerges as a major player controlling the expression levels of proteins that allow pathogenic bacteria to thrive within the host. The stability of mRNA is dictated by the activity of ribonucleases (RNases) that act either alone or in the presence of small regulatory RNAs (sRNAs) and/or with ancillary proteins. The stability of mRNA also depends on growth stage, environmental cues, or stresses (such as the presence of nutrients, metabolites) as well as cell-density, a phenomenon known as quorum sensing [3, 4].

A variety of posttranscriptional regulatory strategies involve RNases. The cell can directly control global RNA decay by adjusting the levels of RNases [4, 5]. Studies using *Escherichia coli* and *Bacillus subtilis *have given a detailed knowledge about the mechanisms of RNA decay and maturation for Gram-negative and Gram-positive bacteria [6, 7]. For instance, posttranscriptional control mediated by sRNAs and RNases is particularly important as it provides the cell with a means to adapt rapidly to sudden environmental changes and stresses. Moreover, it is energetically less costly as it bypasses the need for new protein synthesis. Additionally, fast removal of the sRNA regulator when the stress is over allows the cell to recover and return to its previous genetic program [1]. The advent of new technologies such as deep-sequencing and tiling arrays revealed a plethora of sRNAs and antisense RNAs (asRNAs) encoded in the bacterial genomes [8–11]. Although their functions and the conditions under which they are expressed are only now starting to be understood, we expect a lot of exciting discoveries in the field of RNA regulation.

In this paper, we will primarily focus on RNase-mediated regulation of virulence gene expression in medically relevant Gram-positive bacteria. Different mechanisms will be presented showing that RNases can either activate or repress gene expression. The involvement of RNases in bacterial antiviral defense, transfer of mobile genetic elements and persistence will be presented. We discuss the genomic context within which RNases are embedded and how the conservation of patterns amongst several genomes can give us insight into their expression. Perspectives on the design of new generation antibiotics targeting several components of the RNA degrading machinery will be underscored.

## 2. The RNA Decay Machinery

**Figure 1:**
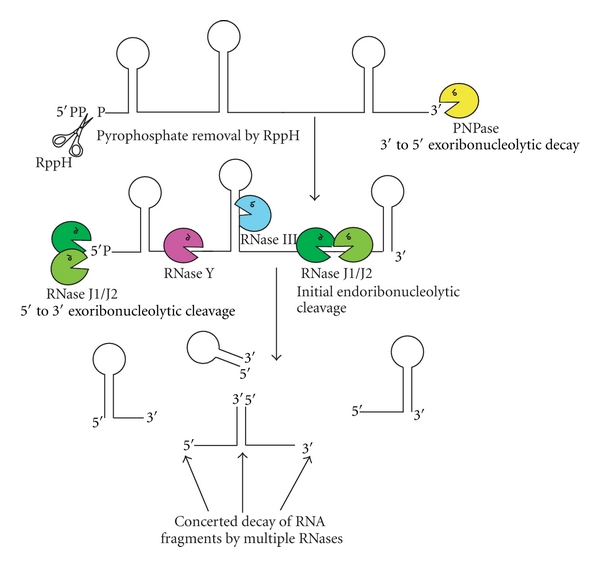
Diagram illustrating RNA decay by the RNases involved in virulence in Gram-positive bacteria. Removal of pyrophosphate from the 5′ end of mRNA by RppH is represented by the scissors. The degradation of RNA via the 3′ end is mediated by the 3′ to 5′ exoribonucleolytic activity of PNPase (in yellow). RNases J1 and J2 (in green) cleave RNA endoribonucleolytically and exoribonucleolytically in the 5′ to 3′ direction. RNase Y (in pink) and the dsRNA-specific RNase III (in blue) cleave RNA endoribonucleolytically.

 Decades of research have resulted in the identification/characterization of several RNases within *B. subtilis*. An exhaustive overview of these is beyond the scope of this paper and we refer readers to some high-quality review articles that cover this topic [5, 7]. Instead, with an emphasis on the most recent discoveries, we will present the works done in *B. subtilis* for specific RNases where homologues in pathogens have been associated with virulence. RNases are broadly divided into two groups: (1) exoribonucleases, which degrade RNA substrates from either the 5′ or 3′end and (2) endoribonucleases that cleave internally within an RNA molecule. The orchestration of mRNA decay in Gram-positive bacteria by the concerted action of several RNases is illustrated schematically in Figure 1.

### 2.1. RNase Y

 Recently, an essential gene within *B. subtilis* (*ymdA*) was determined to be involved with RNA processing and was renamed RNase Y [12, 13]. RNase Y cleaves unpaired regions of RNA and subsequent work demonstrated that RNase Y is the functional equivalent to RNase E in *E. coli, *the major single-strand specific endoribonuclease which initiates RNA processing and degradation [14]. Interestingly, changes in expression levels of RNase Y within *B. subtilis* resulted in altered stability of polycistronic mRNAs required for biofilm formation [15], but this phenotype may be attributed to a polar effect on expression of the downstream gene, *ymdB*, which has been shown to be required for biofilm formation [16]. RNase Y was reported to be involved in riboswitch turnover, as well as to affect global mRNA decay [12, 15]. Recent data from distant bacteria show that RNases such as *E. coli *RNase E and *B. subtilis *RNase Y are not evenly distributed in the cytoplasm but that a fraction is localized at the membrane [17, 18]. Although it is not known whether the localization is a regulated process, the transmembrane domain of RNase Y is essential for the activity of the enzyme *in vivo* [14]. Therefore, these data suggest that RNA turnover is somehow compartmentalized in the cell and that the spatial organization of RNases in bacteria is an additional layer of regulation.

### 2.2. RNases J1 and J2

*B. subtilis* RNase J1/J2 have been the subject of intensive research recently. RNases J1 and J2 were first identified and characterized in *B. subtilis* as the component that endonucleolytically cleaved the *thrS* leader mRNA [19]. Earlier work in *B. subtilis* demonstrated that only RNase J1 is essential for growth [20]. Size exclusion chromatography reveals that recombinant RNase J1 from *B. subtilis* elutes as both a homodimer and a tetramer [21]. RNases J1/J2 are bifunctional and possess both endoribonuclease and 5′–3′ exoribonuclease activities [19, 22]. Additionally, these two proteins can form a heterodimeric complex that has unique cleavage site specificities and efficiency [23].

The exoribonuclease activity of RNase J2 has been shown to be significantly less efficient compared to RNase J1 [23]. The 5′–3′ exonuclease activity, previously not identified in bacteria, appears to be the major function *in vivo* [23]. It has been demonstrated that RNase J1 is involved with global RNA turnover and with processing of 16S and 23S rRNAs [24, 25]. The 5′ triphosphate of primary transcripts and/or the presence of a hairpin structure at the 5′ end protect RNA from degradation by the exoribonuclease activity of RNase J1 [22, 26]. RNases J1/J2 have been found associated with the Gram-positive degradosome complex [13, 27] (discussed below).

The first structure solved for RNase J1 was from *Thermus thermophilus *[26]. The protein consists of three domains, a core *β*-lactamase domain, a *β*-CASP domain, that is specific to members of the *β*-lactamase superfamily of enzymes acting on nucleic acids, and a unique C-terminal domain, that is joined by a flexible linker to the *β*-lactamase domain. Two catalytic zinc (Zn) ions were found in a cleft between the *β*-lactamase and *β*-CASP domains. However, this structure, bound to a UMP substrate, appeared to be in a closed conformation that could not explain the 5′ monophosphate substrate preference nor did it reveal the mechanism for the dual-enzyme activities [26]. The structure of a catalytically inactive mutant form of RNase J1 associated with a 4 nt RNA sequence has recently provided new insights for the mechanisms of the dual enzymatic activities and RNA binding [28]. The binding of the RNA induces a change in the relative position of the three domains with respect to each other as well as specific alterations in conformation of the specific *β*-CASP and *β*-lactamase domains. These movements result in a widening of the narrow catalytic cleft between the domains creating a channel that is wide enough for a single-stranded RNA to enter. The cleaved nucleotide is discharged from the other side through a negatively charged exit tunnel [28]. The binding of RNA causes major rearrangements within the *β*-CASP domain where specific loop regions are displaced by up to 12 Å to accommodate the substrate. The RNA binding pocket consists of positively charged residues that extend beyond the cleavage site, giving a rationale for the binding of longer RNA transcripts and the endoribonucleolytic activity. The recently solved structure and modeling of *B. subtilis* RNase J1 has revealed a similar pattern of conformational changes upon substrate binding [21].

### 2.3. PNPase

 In *B. subtilis, *the nonessential multifunctional PNPase (77 kDa) is the major exonucleolytic RNase, that forms a trimer to catalyze the 3′ to 5′ phosphorolytic degradation of RNA [29–31]. Additionally, under certain conditions, PNPase is able to add nucleotides to the 3′ end of RNA [32]. The 3′ binding and processivity of PNPase appear to be blocked by strong hairpin secondary structures such as Rho-independent terminators, which are often found at the ends of *B. subtilis* transcripts and the processivity was inhibited by the Not I sequence (GCGGCCGC) [33]. Thus, it is believed that PNPase plays the secondary step in RNA decay and that the degradation of nucleotides only occurs after the 3′ end becomes exposed due to an endonucleolytic cleavage by another RNase (RNase III, RNase Y, or RNase J1/J2) [7].

 It is interesting to mention some of the remarkable new functions discovered involving this versatile protein. Recently, PNPase was found to copurify with *B. subtilis* RecN and this complex was able to degrade single-stranded DNA (ssDNA) *in vitro* [34]. The exonucleolytic activity of PNPase on ssDNA was characterized and a functional role for PNPase in homologous DNA recombination in *B. subtilis *was identified [34]. Extending from this work the same group was able to show that *B. subtilis *PNPase catalyzes template-independent polymerization of dNDPs onto 3′ ends of ssDNA [35]. This work has led to the establishment of a molecular model for the role of PNPase in DNA repair [35].

### 2.4. RNase III

 Bacterial RNase III belongs to the Class I RNase III family of enzymes, while Classes II and III include the eukaryotic Drosha and Dicer, respectively, which are involved in biogenesis of siRNA/miRNA in higher organisms (reviewed in [3, 36, 37]). The bacterial RNase III is the smallest of RNase III family members consisting of a catalytic and a dsRNA binding domain [38]. It functions as a homodimer with dimerization occurring through the ribonuclease domains [39]. Recently, a fourth class of RNase III enzymes was found with the discovery of the endoribonuclease Mini III, which is involved with the final steps of maturation of 23S rRNA. This enzyme lacks three out of four dsRNA binding domains and consists of only the catalytic domain [40]. The structures of bacterial as well as eukaryotic RNase III enzymes have helped in understanding their functions (reviewed in [36]).

 RNase III is a Mg^2+^-dependent double-strand-specific endoribonuclease capable of cleaving dsRNA. It recognizes a variety of structures such as imperfect duplexes, helices interrupted by bulged residues, kissing loops, and stacked helices [41–44]. Cleavage by RNase III produces the characteristic type of dsRNA with a 5′ phosphate and a 3′ hydroxyl group and a 2 nt 3′-overhang (reviewed in [5–7]). RNase III is involved in the maturation of large ribosomal RNAs in *E. coli* and *B. subtilis* [45] as well as in the regulation of single and polycistronic mRNAs [5–7, 46]. Moreover, it is involved in the maturation of other housekeeping RNAs such as the small cytoplasmic RNA (scRNA) precursor in *B. subtilis *[47–49]. No consensus sequence motif has been defined for this enzyme but “antideterminants” have been proposed to prevent the recognition of RNA molecules by *E. coli *RNase III [50].

### 2.5. RNase P

 RNase P is a ribonucleoprotein particle that contains at least one protein subunit and a single-ribozyme subunit [51]. In *B. subtilis, *RNase P has been shown to cleave precursor tRNAs and tmRNA forming the mature 5′ end and to cleave the adenine riboswitch which stabilizes the downstream mRNA transcript [52, 53]. The catalytic site is located in the RNA subunit [54]. Crystal structures of RNase P either alone [55] or bound to tRNA [56] reveal that the RNA-RNA recognition occurs through shape complementarity and conserved intermolecular contacts. The active site structure and the conserved RNase P-tRNA contacts suggest a universal mechanism for catalysis. Since biochemical and structural mechanisms for recognition and cleavage of RNA substrates by RNase P have been characterized in-depth by several groups, we refer the readers to an excellent recent review for a comprehensive summary of this work [57].

### 2.6. RppH

 In *E. coli,* RNA pyrophosphohydrolase (RppH) removes a pyrophosphate from the 5′ end of RNA, converting the triphosphate into a monophosphate, a reaction that occurs before RNA degradation by RNase E [58]. The finding that a 5′ triphosphate on RNA is a poor substrate for RNase J1/2 in *B. subtilis* [19] inspired Richards et al. [59] to search for the functional RppH homologues in *B. subtilis.* Bioinformatic searches identified six putative homologues with canonical Nudix hydrolase motifs. To identify the functional RppH, these proteins were expressed and assayed for the deprotection event *in vitro*. They identified an RppH-dependent *B. subtilis* transcript by constructing a ∆*rrpH* strain and screening for mRNAs with altered half-lives [59]. Detailed study of the processing of a polycistronic mRNA reveals that RppH converts the 5′ triphosphate to a monophosphate mRNA prior to degradation by RNase J1. Even though the RppH has not yet been implicated in virulence, this discovery reveals an additional stage in the elegant orchestration of RNA decay. This mechanism is reminiscent of the decapping step that triggers mRNA degradation in eukaryotes [60].

### 2.7. The Existence of a Degradosome in Gram-Positive Bacteria

The degradosome has been first discovered and characterized in *E. coli* [61–63]. A degradosome complex within Gram-positive bacteria has remained elusive until recently [13]. While screening for *in vivo* interaction partners of glycolytic enzymes within *B. subtilis*, several proteins known to be involved with RNA processing and degradation were identified [13]. The primary protein-protein interactions were further evaluated using the bacterial two-hybrid approach. The Gram-positive degradosome consists of three RNases (RNase J1/J2, RNase Y), polynucleotide phosphorylase (PnpA) and two glycolytic enzymes (phosphofructokinase and enolase) [13]. Further characterization identified a DEAD box RNA helicase (CshA) that interacts with RNase Y and PnpA [27]. This helicase was predicted to be localized at the membrane since the protein carries a N-terminal transmembrane domain similar to the sequence present on RNase Y [27]. Recently, the degradosome from *S. aureus* has been characterized and evaluated using the bacterial two-hybrid approach [64]. Although this work confirmed the conservation of the *B. subtilis *multienzymatic complex, different partner interactions were described and additionally, the association of the protein subunit of ribonuclease P (RnpA) with CshA was demonstrated [64].

### 2.8. Genomic Context of RNases

**Figure 2:**
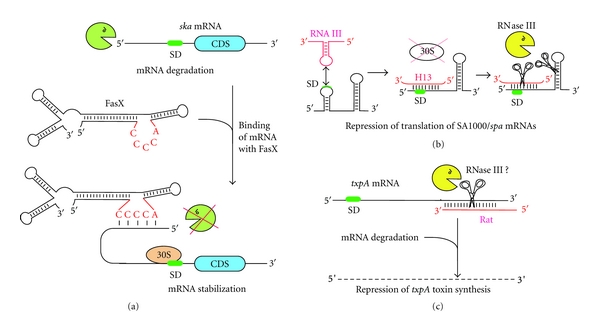
Diagram illustrating different mechanisms of posttranscriptional regulation. (a) Stabilization of *S. pyogenes ska* mRNA. Transcript levels of *ska *are regulated by RNase-mediated decay. Under stress conditions, the FasX sRNA is expressed and binds to the 5′ leader region of *ska* mRNA inhibiting RNase degradation [70]. (b) *S. aureus *RNAIII/RNase III repression of translation. The quorum-sensing regulatory RNAIII uses a regulatory hairpin to bind to *spa* mRNA (or SA1000) target sequence. The initial loop-loop interaction is converted to a duplex sequestering the Shine-Dalgarno sequence (SD). Access of the ribosome is blocked and translation is repressed. RNase III is recruited to the hybrid region and to an additional hairpin present in *spa* and cleaves the transcript making the regulatory event irreversible [71, 72]. (c) Repression of *B. subtilis *TxpA toxin synthesis. The asRNA Rat is transcribed convergently and is fully complementary to the *txpA* mRNA. Binding of Rat asRNA to *txpA* transcript induces rapid degradation of the mRNA [73]. The RNase performing the initial cleavage might be RNase III although the nature of the enzyme and the structure of the hybrid are not yet determined.

**Figure 3:**
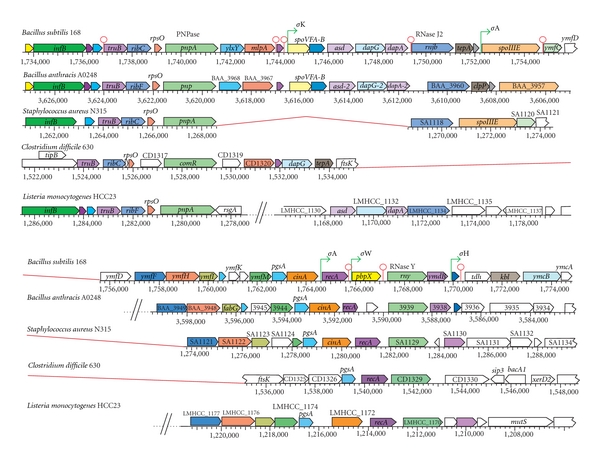
Alignment of the genomic context for three components of the RNA degradosome. Homologues of PNPase, RNase J2, and RNase Y were aligned from the different Gram-positive organisms, *Bacillus subtilis *168, *Bacillus anthracis *A0248, *Staphylococcus aureus *N315, *Clostridium difficile* 630, and *Listeria monocytogenes* HCC23. Alignments were made using the multigenome alignment tool on http://www.biocyc.org/. Red lines indicate directly adjacent regions of the chromosome and the diagonal lines indicate a chromosomal gap. Genes that are conserved in bacteria are shown with the same color.

 The importance of genomic organization and operon structure for gene expression has been well documented [65]. Often genes with similar function or associated to the same biosynthetic pathway tend to be found within the same operon or in close proximity on the chromosome. Combining genomic information from several public databases (NCBI, genolist, and Biocyc.org), we analyzed the conservation of genetic organization across four families of pathogenic Gram-positive bacteria additionally including *B. subtilis*. Using the multigenome alignment tool (http://www.biocyc.org/), the genomic context was aligned for several Gram-positive organisms (see Figures 1–8  in Supplementary material available online at doi: 10.1155/2012/592196). Some of the most noteworthy features are highlighted below. 

Several of the genes (*pnpA*, *rnjB*, *rny*) for the proteins involved in the degradosome are located in a relative close proximity to each other on the chromosome and genetic arrangements appear to have evolved in Gram-positive species (Figure 3). However, a loose conservation of *pnpA-rnjB-rny* gene cluster is observed among the genomes. Interestingly, the presence of *rpsO* upstream of *pnpA* is found in all genomes and in *E. coli *as well, suggesting that *pnpA* and *rpsO* are ancient genes of a common origin. In *B. subtilis*, *pnpA* is part of a polycistronic operon located 8 kb upstream of *rnjB*, and the *rnjB *operon is sandwiched between two chromosomal regions that contain several sporulation genes. In *S. aureus*, *pnpA* and *rnjB* are located directly next to each other (Figure 3). In *Clostridia* species, no RNase J2 homologue was found, but a large genomic rearrangement has brought *pnpA* relatively close to *rny*. In *Listeria* species, a very different genomic context is observed, where large genomic insertions separate the genes of this cluster. The proximity of *pnpA-rnjB-rny* locus within several Gram-positive organisms may have functional consequences. For instance, located within the same locus are certain genes that have been identified to cross-regulate expression and activity. The enzymatic activity of PNPase on single-stranded DNA is in part modulated by RecA, a gene located upstream of *rny* [35]. Decay of the highly conserved *rpsO* mRNA is initiated by RNase Y followed by PNPase [66]. Recently, in *Streptomyces coelicolor, *it has been shown that transcripts originating at the *rpsO* promoter read-through into *pnpA* and become processed by RNase III [67].

The clustering of RNase genes, along with several genes for sporulation, cell wall peptidoglycan biosynthesis, motility, and cell division are also noteworthy. In *E. faecalis*, cotranscribed with *rnjB* are two genes involved with cell wall biosynthesis and *rnjB* has been shown to regulate pili formation at the posttranscriptional level [68]. In *B. subtilis*, RNase Y (*rny*/*ymdA*) is the first gene of a bicistronic operon including *ymdB* which was recently shown to play a critical role in the bistable expression of genes involved in flagella and biofilm formation [16]. Moreover, the genes located upstream of *rny*, *pgsA* (cell wall biosynthesis), *cinA *(competence-damage inducible regulator), and *recA* (member of SOS regulon) are relatively conserved among Gram-positive bacteria. The gene encoding CshA, the RNA helicase of the degradosome in *B. subtilis *[27], is also part of an operon including two genes encoding proteins involved in peptidoglycan biosynthesis. The genomic context of this operon is conserved in other species (see Supplementary Figure 8). RNase III (*rnc*) is transcribed as the first of a three-gene operon in *B. subtilis,* also encoding the essential *smc* (chromosome condensation and segregation ATPase) and *ftsY *(signal recognition particle) [69]. This organization is also highly conserved in all Gram-positive species (see Supplementary Figure 2). 

Transcriptomic array data for *S. aureus* [74, 75] reveal that all RNases are expressed in vegetative growth. At the transition to stationary phase, the levels either (i) drop dramatically and remain low thereafter for RNase J2, RNase Y, and RNase P or (ii) reduce slightly only to catch back later into stationary phase for RNase J1, PNPase, and RNase III. The reduced transcript levels of several *S. aureus *RNases in stationary phase [74, 75] appear to correlate with the demand for peptidoglycan/ribosome biosynthesis.

## 3. Ribonucleases Acting in Virulence

### 3.1. Global Roles of RNases in Virulence

 The role of several RNases in virulence has been recently studied in two major pathogenic bacteria,* Streptococcus pyogenes *and* Staphylococcus aureus*.* S. pyogenes* (group A *Streptococci*, GAS) and *S. aureus* cause mild to systemic and life threatening diseases. The emergence of methicillin resistant *S. aureus* strains acquired both in hospitals and, in the community, has resulted in more deaths annually than HIV in the United States in recent years [76, 77].

 GAS transcripts have been recently classified into two Classes, I and II, depending on their stability during stationary phase of growth [78]. Class I transcripts appear very labile during stationary phase, while Class II transcripts encoding several virulence factors such as *sagA* (streptolysin S), *sda* (DNase), and *arc* (arginine deiminase) have prolonged half-lives. It has been shown that the 3′–5′ exonucleolytic activity of PNPase is responsible for degrading substrates of class II after an elongated lag phase where mRNAs are stable [79]. The initial endoribonucleolytic cleavage of Classes I and II transcripts is mediated by RNases J1 and J2, which are essential in GAS [78, 80]. It was proposed that Class I transcripts are better substrates for RNases J1 and J2 and only after their depletion, degradation of Class II transcripts is initiated. The amounts of RNases J1, J2, and PNPase as well as putative signals they respond to, might be critical for such growth phase-dependent regulation.

The roles of ribonucleases in virulence gene expression are summarized in Table 1. RNase Y, encoded by *cvfA* in *Streptococcus* and *Staphylococcus*, has been shown to affect virulence in both pathogenic bacteria in silkworm and murine models [81–83]. In *S. pyogenes*, a *cvfA* deletion affected expression of several virulence factors [82]. Moreover, microarray analysis revealed differential expression of 29% of genes indicative of the importance of RNase Y in the initiation of mRNA decay [82]. These data suggested that RNase Y mediated downregulation of metabolism and upregulation of certain virulence factors facilitates the acquisition of cellular components from the host. The effect of RNase Y on virulence gene expression occurred mainly in stationary phase [82] in agreement with data showing that virulence factor expression in *S. pyogenes* is strictly dependent on growth phase [84]. RNase Y-mediated regulation was shown to be dependent on the nutritional status of the cells implying that the enzyme is involved is sensing the nutrient availability (directly or indirectly) [82]. However, its deletion did not affect the ppGpp levels, therefore the observed effects were not linked to stringent response [82]. *S. pyogenes* RNase Y interacts with the glycolytic enzyme enolase [82] which might provide a link between nutritional status and RNase Y-mediated gene expression. RNase Y has been reported to affect production of virulence factors in *S. aureus* either through expression of the accessory gene regulatory locus, *agr* or independently [83]. Biochemical analyses revealed that the predicted phosphohydrolase domain (HD domain) of RNase Y possesses a phosphodiesterase activity against 2′, 3′-cyclic nucleotide and this activity is required for virulence [83]. RNase Y was predicted to contain a transmembrane domain in *S. pyogenes* and *S. aureus* [82, 83], similar to the *B. subtilis* enzyme [17].

Studies have indicated that the capacity of *S. aureus* to differentially express subsets of virulence factors according to stress and growth phase is largely attributed to mRNA stability [4]. In particular, it was shown that mRNA stabilization occurs in stationary phase as well as under cold-, heat-, acid-, and alkaline-shock and the stringent response [85, 86]. The authors showed that PNPase affected bulk mRNA decay and was shown to be essential for cold-growth [4] as is the case for *E. coli, Salmonella enteric,* and several *Yersinia *species (reviewed in [87]). In addition, RNase P was also described as a major RNase involved in bulk mRNA degradation that has direct consequences on the expression of virulence factors at the stationary phase of growth [75]. Interestingly, *rnpA*-depleted cells exhibited attenuated virulence in a mouse infection model [75].

### 3.2. RNases and Mechanisms of Regulation of Specific Genes in Pathogenic Bacteria

#### 3.2.1. sRNA and RNases Activating Gene Expression

The activation of an S. pyogenes mRNA encoding a virulence factor (ska, streptokinase) depends on the presence of a sRNA, FasX [70]. FasX binds to ska mRNA 30 nts upstream of the AUG and forms a 7 nts-long helix. This double-stranded structure leads to an increased stability of the mRNA (Figure 2(a)). Notably, a C-rich sequence motif present on FasX is employed for the interaction, similarly to what has been described for *S. aureus* RNAIII and other sRNAs [71, 88]. Deletion of either RNase Y or PNPase did not result in stabilization of *ska *mRNA indicating that FasX-dependent stabilization is due to a limited access of RNases at the 5′-end [70]. Whether the 5′-3′ exoribonucleolytic property of RNases J1 and J2 is responsible for *ska *mRNA degradation, remains to be tested.

Within *Clostridium perfringens*, the VirR/VirS two-component system regulates several virulence genes including transcription of the 386 nts sRNA, VR-RNA [89]. Recent work by Obana et al. [90] has shed light onto the VR-RNA-dependent regulation of the toxin collagenase (*colA*). Base pairing of VR-RNA with the 5′UTR of *colA* mRNA induces cleavage by an unknown RNase immediately downstream of the *colA*-VR-RNA duplex. This processing in turn leads to the formation of a shorter hairpin and increased mRNA stability *in vivo*. Mutational analysis of the RBS indicates that ribosome binding to the processed mRNA additionally stabilizes the *colA* mRNA [90].

 Hence, the secondary structure of mRNA has an important role in controlling transcript stability as highlighted by the two examples for *ska* and *colA* mRNAs [70, 90]. Interestingly we have shown that in *S. aureus*, RNase III mediates transcript stabilization of *cspA *mRNA, encoding a cold-shock protein, through processing of a long hairpin structure in the 5′UTR. This processing leads to the formation of a short but stable hairpin that enhances the stability of the mRNA and its translation (Lioliou et al., submitted).

*Enterococcus faecalis* is an opportunistic Gram-positive responsible for many nosocomial infections [91]. Recently, Gao et al. [68] showed that the *E. faecalis rnjB, *encoding RNase J2, is involved in the regulation of pilus gene expression and biofilm formation. Pili expression is important for pathogenicity of many Gram-positive organisms [92]. Inactivation of *rnjB* results in destabilization of *ebp* operon transcript encoding the pilus proteins [68]. The mechanism of action by which RNase J2 stabilizes the mRNA and whether this regulation takes place in other Gram-positive bacteria producing pili remains to be shown.

#### 3.2.2. sRNA and RNases Repressing Gene Expression

 The effect of RNase III in virulence in *S. aureus* has been well documented. Specifically, in stationary phase, RNase III acts together with the *agr* encoded regulatory RNAIII [93] to repress the expression of several adhesin factors and the repressor of toxins, Rot [44, 71, 72]. RNAIII is a multifunctional RNA that encodes a virulence factor, hemolysin delta. Through its 3′ UTR, RNAIII interacts with mRNAs either by forming imperfect duplexes or by promoting the formation of loop-loop interactions. In cases where translational repression occurs, the Shine-Dalgarno is sequestered and ribosome binding is prevented. RNAIII-mRNA complexes in turn constitute targets for RNase III cleavage to render regulation irreversible (Figure 2(b)). Translational repression of Rot indirectly results in the activation of toxins and in the repression of adherence factors. As more functions of regulatory RNAs are unveiled [8, 88, 94–96], it is predicted that other sRNAs, such as the group I toxin-antitoxin systems, might act coordinately with RNase III to regulate gene expression. In a recent report, it was shown that a Δ*rnc* strain was attenuated in a murine infection model, and that RNase III is involved in the regulation of extracellular protein secretion, such as the extracellular fibrinogen binding protein Efb in *S. aureus* [97]. This was achieved through regulation of the levels of *secY2* mRNA encoding a protein of the accessory secretory system.

 In Gram-negative bacteria, Hfq has been shown to be a critical component of sRNA-mediated regulation [98]. Evidence of a similar role for Hfq in Gram-positive bacteria has remained elusive until recently [99]. Work by Nielsen et al. [100] showed that within *Listeria monocytogenes*, translational regulation of lmo0850 mRNA mediated by the binding of LhrA sRNA is dependent on Hfq. The specific RNases involved in the degradation of the lmo0850 transcript still remain to be determined. Additionally, a *lhrA* deletion within *L. monocytogenes* altered the expression levels of approximately 300 genes, including *chiA*, a known virulence factor that encodes a chitinase [101].

#### 3.2.3. RNase and Defense Mechanism: CRISPR

Recently, the role of RNase III in bacterial immunity against phages and plasmids has been demonstrated [102]. CRISPR/Cas (clustered, regularly interspaced short palindromic repeats/CRISP-associated proteins) systems mediate bacterial immunity against foreign invading DNA, such as plasmid and phages. The CRISPR genetic loci encode for spacer-repeat sequences as well as their associated Cas endoribonucleases. The repeat sequence is usually identical and can be found between 2–249 times within a locus. The spacer sequences are unique and originate from phage or plasmid sequences that have been integrated in the genome and which subsequently confer immunity against the specific phage or against plasmid conjugation [103–107]. The CRISPR loci are transcribed as a long pre-crRNA, which is then matured into small RNAs (crRNA) consisting of a single spacer-repeat unit, which effectively attacks foreign DNA. Processing of pre-crRNA into crRNAs is usually performed by the Cas endoribonucleases. However, some Cas homologues are absent from certain subtypes of CRISPR systems as in *S. pyogenes*, which lacks three Cas proteins, Cse3 (CasE), Cas6, and Csy4. In these cases, the host-encoded RNase III mediates maturation of the pre-crRNA acting in concert with a *trans*-encoded RNA (*trans*-activating CRISPR RNA, tracrRNA) and the endoribonuclease Csn1 [102]. Particularly, it has been reported that tracrRNA interacts with almost perfect complementarity with the repeat units in the pre-crRNA. In a first processing event, RNase III cleaves specifically the duplexes and subsequently, a second cleavage occurs in the spacer sequence. The exact mechanism underlying the second processing event and whether it is carried out by Csn1 remains to be elucidated. Csn1 also aids the interaction of the two RNAs and possibly stabilizes the tracrRNA. This type of regulation was found conserved in other bacteria such as *Listeria innocua, Neisseria meningitides, Streptococcus mutans,* and *Streptococcus thermophilus*. Based on examination of several bacterial genomes, it was suggested that tracrRNA possibly co-evolved with the repeat sequences of crRNA [102].

#### 3.2.4. Unusual RNases: Riboswitches with Catalytic Activities

Riboswitches are *cis*-acting regulatory RNA sequences that control expression of downstream genes by binding metabolites that induce structural changes to the transcript [108]. An interesting study recently highlighted the regulatory importance of cyclic-di-GMP [109]. In this study, a new class of c-di-GMP riboswitch was identified that is linked in tandem to an allosteric self-splicing ribozyme upstream of a putative virulence gene within the genome of *Clostridium difficile*. Binding of c-di-GMP to this RNA structural motif induces folding changes at the splice site causing a different splicing pattern. The binding of c-di-GMP results in the inclusion of a perfect RBS directly upstream of a nonconventional UUG translational start codon. Without the second messenger signal, c-di-GMP, the final spliced mRNA lacks the RBS and is not translated [110]. This work illustrates a new level of posttranscriptional complexity within bacteria.

 The discovery of the mechanism for transcript destabilization induced by the *glmS* ribozyme has demonstrated how the cell can sense nutritional status and modulate gene expression accordingly using catalytic RNA cleavage followed by RNase-mediated decay [111]. In *B. subtilis*, glucosamine-6-phosphate binds to the *glmS* ribozyme stimulating site-specific RNA self-cleavage *in vivo*. This cleavage results in transcripts that contain a 2′-3′ cyclic phosphate at the 3′ end and a hydroxyl group at the 5′ end. After cleavage, the downstream transcript is rapidly degraded. It was shown that targeting of *glmS* RNA for degradation was due to the 5′ hydroxyl end which was a substrate for the 5′–3′ exoribonuclease activity of RNase J1 [111]. This work demonstrates that metabolite-sensing ribozymes enable the cell an efficient means to respond to their environment.

#### 3.2.5. Toxin-Antitoxin Systems and Stress Response

Toxin-antitoxin (TA) systems are continuously being discovered across bacterial species. Their involvement in phage resistance, plasmid maintenance, stress responses, and bacterial persistence is well documented (for reviews, see [112–118]). These systems are classified into three main types. In the first one, the antitoxin is a noncoding RNA (ncRNA), which is antisense to the mRNA encoding the toxin. For type II, both toxin and antitoxin are proteins. Type III (ToxIN) was recently discovered and employs an antitoxic sRNA, which binds and inhibits a protein toxin. For type II and III systems, the toxin and the antitoxin are cotranscribed as part of an operon, whereas for type I systems the two genes are encoded on the opposite strands overlapping in their 5′- or 3′-ends. In all cases the antitoxin, RNA or protein, is labile and subject to degradation, while the toxin is stable. Upon conditions that favor elimination of the antitoxin, the TA complex is disrupted, and the toxin is released (or translated) to exert its toxic effect. Type I toxins usually consist of small hydrophobic peptides, the translation of which is turned off by antisense RNAs (asRNAs) [116]. In the case of *E. coli hok*/Sok and *tisAB*/IstR systems, RNase III is the key enzyme which degrades the mRNA-asRNA complex [118]. Toxins of type II act usually as endoribonucleases (MazF and RelE) or inhibit DNA gyrase (CcdB) [112–114]. The *E. coli* MazF is an endoribonuclease cleaving mRNAs at a defined ACA consensus sequence independently of the ribosome, while RelE is a ribosome-dependent endoribonuclease that cleaves mRNAs positioned at the ribosomal A-site (reviewed in [112, 114]). The unique type III toxin, ToxN, was demonstrated to have endoribonuclease activity *in vitro* and was capable of cleaving its inhibitory antitoxic sRNA [119, 120]. These data suggest that ToxN possibly acts as an RNase to inhibit translation and slow down bacterial growth. The mRNA targets of ToxN remain to be discovered.

The only type II TA system identified in *B. subtilis* so far is *ndoAI*/*ndoA* where *ndoA* encodes the toxin (EndoA, MazF homologue), which cleaves at unpaired UACAU sequences, and *ndoAI* encodes the antitoxin [121, 122]. Depending of the nature of the stress, the TA module in *B. subtilis* can be protective or lethal [123]. The authors hypothesized that this behavior would allow the cell a way to determine if the stress was mild enough to be repaired or so severe as to activate the cell death pathway [123]. Interestingly, a type I TA system encoded in the chromosome of *B. subtilis* was identified [73]. It consists of the TxpA toxin and its asRNA, RatA. The two RNAs are transcribed convergently overlapping at their 3′ ends. Pairing between *txpA*-RatA leads to degradation of the mRNA by an unknown RNase (Figure 2(c)). Given its specificity for dsRNA, RNase III could be a good candidate to mediate the degradation. As the number of asRNAs found in bacterial genomes constantly increases [8–11], RNase III might prove to be an important player in this type of regulation.

 The PemIK TA module from *B. anthracis* has recently been characterized [124]. The PemK was shown to be an endoribonuclease toxin that shares 96% similarity to the EndoA (MazF homologue) toxin from *B. subtilis*. Biochemical characterization of the TA module confirmed that PemI inhibits PemK-mediated endoribonuclease activity. The catalytic residues in PemK were defined *in vitro*, and surprisingly, the catalytic mutants retained the capacity to bind PemI efficiently [124]. The PemIK interaction was characterized* in vitro* giving clues to the conformational changes that take place following complex formation. Synthetic peptides were designed to disrupt the PemI-PemK interaction and to inhibit the endoribonuclease activity of PemK, demonstrating that TA modules can be potential antimicrobial targets [124].

The identification of MazEF and to a smaller extent *axe-txe*, *relBE,* and **ε*-*ζ**homologues in plasmids of vancomycin resistance *enterococci* (VRE) isolated from patients [125] demonstrates their clinical importance. The authors showed that the MazEF system was transcribed and endowed *enterococci* with plasmid stability. Moreover, the *mazEF* genes were located on the same plasmid with the vancomycin resistance gene cluster, *vanA*. Recently, the *axe-txe* system was identified in a plasmid from *Enterococcus faecium*, which also encoded multiple drug resistances. The system was expressed in clinical isolates and Txe was shown to have endoribonucleolytic activity *in vivo* [126]. The presence of these systems in enterococcal plasmids might imply a role in the transfer of antibiotic resistance genes to other species such as MRSA [127] linking TA systems to development of virulence.

MazEF was also identified in *S. aureus* and the unpaired UACAU recognition sequence for endoribonucleolytic cleavage was established [128, 129]. Induction of MazEF leads to destabilization of *sraP* mRNA which contains the consensus cleavage motif. The occurrence of the consensus sequence within coding sequences of other virulence factors was also demonstrated [129]. The induction of the MazEF had an effect on the expression of *spa* and *hla* mRNAs [130]. As *mazEF* and *sigB* are transcriptionally linked, *sigB* expression is partially dependent on factors affecting transcription of *mazEF* such as heat-shock and antibiotic stresses [131]. SigB was also shown to downregulate expression of *mazEF*, therefore creating a feedback inhibitory loop that possibly affects its own expression [131]. These findings have significant implications in mediation of stress responses and virulence as SigB is a major regulator of these biological processes in *S. aureus* [132]. Recently, the expression of a chromosomally encoded MazEF system in clinical isolates of MRSA strains was reported [133]. Therefore, this TA module is an important player in regulation of pathogenicity and might constitute a novel target for antibiotics.

Interestingly, it was recently shown that *E. coli *MazF cleaves preferentially ACA sequences in mRNAs located close by to the AUG codon thus generating leaderless mRNAs [134]. In addition, the enzyme also cleaves 16S rRNA within the 30S subunit and removes its last 43 nucleotides including the anti-Shine and Dalgarno sequence. This in turn creates a subpopulation of ribosomes that are able to translate the leaderless mRNAs [134]. Whether the formation of such specialized ribosomes produced under stress conditions can be generalized to all bacteria remains to be addressed.

*Mycobacterium tuberculosis* is a major worldwide health problem and in 2009 approximately 1.7 million people were estimated to have died from tuberculosis (WHO, Global tuberculosis control report 2011, WHO/HTM/TB/2011.16). Bioinformatic analyses revealed that *M. tuberculosis* encodes 88 putative TA systems, 30 of which were shown to be functional [135]. Remarkably, most of the TA systems were conserved within the *M. tuberculosis* complex (MTBC) while they were absent from closely related species. This implies that they were acquired after the speciation event and that they probably play a significant role in pathogenicity. The VapBC is the most abundant TA module in *M. tuberculosis* represented by 47 members. Subsets of VapBC modules were shown to be toxic when expressed and conversely this toxicity was counteracted by coexpression of the cognate VapB antitoxin [135, 136]. VapC functions as an RNase *in vitro* and this may account for the inhibition of translation observed *in vivo* which profoundly affects gene expression in response to different environments and stresses [135, 136]. Certain sets of TA systems were found upregulated under hypoxia and during infection of macrophages [135]. Previously, it was shown that the recognition sequences of MazF-mtb were less frequently present in proteins associated with pathogenicity (reviewed in [113]). Hence, certain TA modules can direct degradation of specific mRNAs in response to stress rather than inhibit bulk translation.

Interestingly, Winther and Gerdes [137] have shown that enteric VapC encodes a tRNase that cleaves initiator tRNA^fMet^ between the anticodon stem and loop. Cellular depletion of tRNA^fMet^ had a bacteriostatic effect on cultures and production of VapB allowed cells to resume growth. The depletion of tRNA^fMet^ by VapC not only inhibited cell growth but was additionally found to activate initiation of translation at correctly positioned elongator codons. The authors further speculated that this mechanism has the potential to translate reading frames that were normally silent. This work in enteric bacteria has significant implications for virulence in Gram-positive organisms, particularly *M. tuberculosis* where VapBC modules are highly represented [135, 136]. The oxidative burst within macrophages generates superoxide anion (O_2_^−^) and singlet oxygen, which can be lethal to cells [138]. A mechanism to globally reduce translation, such as cleavage of tRNA^fMet^, would reduce the occurrence of toxic events and enhance survivability of the organism.

 Bacterial persistence is a phenotype where part of the cell population enters a dormant nongrowing state which in turn confers resistance to antibiotics and other stresses [139]. The involvement of TA systems in development of persistence in *E. coli* was recently reported [140]. The authors showed that overexpression of the toxin led to the persister phenotype and that successive deletion of all TA systems in *E. coli* resulted in a dramatic decrease in formation of persisters [140]. Consistent with involvement of TA systems in the persistence phenotype, the transcriptome of persister *M. tuberculosis* revealed overexpression of TA systems [141].

The importance of the CRISPR systems in antiphage resistance was discussed earlier. Abortive infection (Abi) is mediated by the type III ToxIN systems and constitutes another mechanism by which bacteria defend against phages [142]. During Abi, a phage-infected bacterium altruistically commits suicide to prevent the spread of phage within the population. ToxIN systems were identified in the Gram-negative phytopathogen *Erwinia carotovora* but homologues are found in the genomes of several Gram-positive and Gram-negative pathogenic bacteria [120]. The *hok*/Sok (type I), MazEF (type II) systems have also been shown to confer phage resistance to their hosts (reviewed in [112, 118]). This type of antiviral immune system could be critical for limiting horizontal transfer of phage encoded virulence factors between pathogenic species.

## 4. Regulation of RNase Activity and RNA Stability: Emerging Issues

### 4.1. RNases and sRNA-Dependent Regulation

RNases often act in concert with sRNAs and/or RNA binding proteins. Interaction with a sRNA can lead to occlusion of the ribosome binding to repress translation. Often an RNase is recruited to the site of interaction to degrade the mRNA making the regulation irreversible. It has also been proposed that in the absence of ribosome binding, the mRNA becomes more exposed to the action of RNases so that repression of translation can lead to rapid degradation [3, 5]. Therefore, sRNAs are key players in fine-tuning mRNA levels. Most of our knowledge on sRNA stability comes from Gram-negative bacteria. In those cases examined, RNase III, PNPase, and RNase E are involved in the degradation of the sRNA, which can be coupled or uncoupled to that of the mRNA target [143–146]. Hfq is also an important player which protects the sRNAs against degradation [98]. Unexpectedly, sRNAs were recently shown to be destabilized in *E. coli pnpA* mutant cells in exponential phase of growth. It was proposed that PNPase protects sRNAs from degradation mediated by RNase E [144]. While *S. aureus *RNase III has been shown to initiate the decay of mRNAs repressed by the quorum sensing dependent RNAIII [87], little is known on the roles of RNases associated with the sRNA-dependent regulation in Gram-positive bacteria.

In the cases described so far where regulation of mRNA stability occurs through the combined action of a sRNA and a ribonuclease, the initial cleavage site is located within the mRNA-sRNA duplex as it is the case for RNase III [99] or proximal to the base-pairing region as exemplified for RNase E [1, 6]. A novel mode of action for *E. coli* RNase E was recently reported where the enzyme acts at a distance cleaving further into the coding sequence [147]. RNase E was found tethered to the RBS of *sodB* mRNA through association with Hfq and the sRNA RyhB which in turn occluded ribosome loading onto the RBS. When the mRNA was stripped of translating ribosomes, RNase E cleavage sites present within the coding sequence were exposed to the endonucleolytic activity of RNase E. Whether a similar mechanism exists in Gram-positive bacteria, remains to be elucidated.

Hfq, although a major player in sRNA-mediated degradation in Gram-negative bacteria [98], does not seem to have such an important role in RNA decay in Gram-positive bacteria with one exception reported so far (see earlier). Other RNA binding proteins may carry out the role of Hfq. Interestingly, a conserved protein was found in *Sinorhizobium meliloti,* SMc01113/YbeY, which shares structural similarities with the MID domain of the Argonaute (AGO) proteins. Deletion of this protein induces pleiotropic effects as found for Hfq. Moreover, the protein regulates the accumulation of sRNAs and mRNAs similarly to Hfq [148]. This protein is conserved in Gram-positive bacteria although its function has not yet been studied in these organisms. In *B. subtilis*, three small basic proteins were proposed to act as RNA chaperones acting in concert with the sRNA FsrA, to promote degradation of transcripts encoding iron-using proteins under conditions of iron deprivation [149].

Hence, these examples illustrate the important role of RNases in mRNA turnover and gene regulation and show that repression of translation mediated by sRNA (also by translational repressor proteins) is often subsequently followed by mRNA degradation. All these regulatory events involve the catalytic activity of the enzymes. However, one cannot exclude that RNases might also regulate gene expression solely through their RNA binding activity. Notably, the dsRNA binding activity of RNase III has been reported to promote translation of lambda phage cIII RNA [150].

### 4.2. Modification of the Degradosome

 The activity of RNases or other enzymes of the degradosome can be modulated. The first evidence of a modified degradosome came during a screen for suppressors of a cold-sensitive phenotype in a *csdA* mutant [151]. It was demonstrated that the RNA helicase, *csdA*, becomes incorporated in the RNA degradosome complex in* E. coli* after cold shock [151] and it can functionally replace RhlB, the typical RNA helicase within the degradosome. It was proposed that the cold shock-induced CsdA associates with the degradosome to facilitate the unwinding of structured RNAs at cold temperatures. Furthermore, recent evidence revealed the association of the degradosome with components of central metabolism suggesting that these modified complexes may be part of a feedback network that allows the cell to coordinate RNA decay with metabolic conditions. In *Caulobacter crescentus,* the Krebs cycle enzyme aconitase was found associated with the degradosome as opposed to the glycolytic enzyme enolase that is found in *E. coli* [152]. Binding of aconitase to the complex occurred through interaction with RNase E and levels of RNase E varied during the cell cycle. The connection between RNA decay and central metabolism was further illustrated by the fact that citrate, a metabolite of the Krebs cycle, has an inhibitory effect on the activity of PNPase in *E. coli* [153]. It was recently pinpointed that an oxygen sensing system is able to adjust the level of c-di-GMP available to PNPase within a large ribonucleoprotein complex in *E. coli* and that this enhances PNPase activity [154]. Within this complex, PNPase, enolase, RNase E, RNA terminator protein Rho, several chaperone proteins (DnaJ, DnaK, GroEL), and oxygen-sensing proteins (DosC and DosP) were identified in addition to an RNA moiety [154]. Altogether these different studies have identified variations to the degradosome complex that confer to the cell the capacity to sense and adapt to environmental changes. It is expected that future work characterizing modifications of the degradosome complex and their impact on RNA decay will uncover connections between RNA regulation and global metabolism.

 Moreover, several examples have been highlighted where the activity of RNases can be cross-regulated. For instance, *E. coli *PNPase synthesis is autoregulated at the posttranscriptional level by an RNase III-dependent mechanism [155]. In fact, many of the RNases also appear to be regulated by a feedback mechanism and due to the fact that they are involved in rRNA processing, their expression is thus coordinated with ribosome synthesis [156–158]. Moreover, a protein regulator has been reported for *E. coli *RNase III [159]. One can envision sRNAs possibly having a role in modulating ribonuclease activity. As underscored above, the genomic context of the RNases might offer valuable cues towards understanding regulation of their expression. Stage of growth and different stresses emerge as common themes in regulation of RNases activity [4, 78]. Surely our appreciation of regulation of RNase activity is far from being complete.

## 5. Conclusions and Perspectives

 This paper highlights the importance of RNases in gene expression in several Gram-positive pathogenic bacteria. Furthermore, we have illustrated by several examples the roles of specific RNases in the regulation of the expression of mRNA targets involved in virulence. However, the full repertoire of targets for RNases, and the roles of accessory proteins (RNA helicases, RNA-binding proteins) in gene regulation still need to be assessed, especially in Gram-positive bacteria. Without doubt our knowledge will greatly advance from current methodologies such as deep sequencing and tiling arrays where the full RNome of a bacterium can be evaluated in wild-type and mutant backgrounds. Moreover, high-throughput sequencing combined with crosslinking immunoprecipitation (HITS-CLIP) has proven valuable for identifying RNA-protein interactions [160, 161]. This method could also lead to identification of the set of target RNAs of an RNase. These new possibilities are expected to offer valuable insights into the function of these enzymes.

 The emergence of antibiotic resistances amongst pathogenic bacteria urgently necessitates the discovery of novel drugs. Several possibilities of targeting components of the RNA decay machinery in the design of novel drugs have been reported. Recently, an inhibitory compound of RnpA was identified that proved to be effective against several strains of MRSA, biofilm associated *S. aureus *and against other Gram-positive pathogens [75]. Despite the fact that the identified molecule exhibited cytotoxicity against a human cell line and cannot be further exploited, it nevertheless sets the foundation for novel antimicrobial strategies targeting the RNA degrading machinery. Riboswitches have also been investigated as possible drug targets. Efforts have been focused on designing ligands nonmetabolizable by the cell that can specifically target riboswitches and repress growth [162, 163]. TA systems have been suggested as good candidates for novel antibacterial strategies. Two modes of action have been envisioned for newly designed drugs aiming at activation of the toxin. The first approach involves turning down production of the antitoxin either at the transcriptional or the translational level whereas the second one aims at the disruption of the toxin-antitoxin interaction. In both cases, the result is degradation of the labile antitoxin and release of the toxin which ultimately results in self-inflicted cell death [164]. The emergence of antibiotic resistances often relies on the presence of resistance genes residing on mobile genetic elements like plasmids [125] which can be transferred to different species [127]. Since TA modules are responsible for stabilization of these elements, a strategy for targeting TA systems seems to hold promise for the design of novel antibiotics.

## 6. Note Added in the Proof

A recent study by Lasa et al. [165] revealed an RNA quality control function of *S. aureus* RNase III. The enzyme eliminates antisense RNA production by specific processing of sense-antisense complexes.

## Supplementary Material

In the supplementary material, the genomic context for the genes encoding the ribonucleases *rnpA, rnc, rnjA/rnjB, pnpA, rny* as well as the pyrophosphohydrolase rppH and the helicase cshA is shown for several major Gram-positive pathogenic bacteria, namely, *Listeria monocytogenes, Staphylococcus aureus, Mycobacterium tuberculosis, Enterococcus faecalis, Clostridium difficile, and Bacillus anthracis*.

## Figures and Tables

**Table 1:** Ribonucleases: mechanism of action and known virulence targets/phenotypes in Gram-positive pathogens.

Ribonuclease (name of the gene)	Mechanism of action	Result	ncRNA or protein partner	Targets in virulence/phenotype/comments	Organism	Refrence
PNPase (PNP)	3′ to 5′ exoribonucleolytic	Repression		Essential for cold stress response/affects global mRNA turnover	*S. aureus*	[4]
RNase P (*rnpA*)	Endoribonucleolytic	Repression		Affects expression of a large set of virulence factors/*rnpA* depleted cells exhibited attenuated virulence in a mouse infection model	*S. aureus*	[75]
RNase III (*rnc) *	dsRNA specific endoribonucleolytic	Repression	RNAIII	Represses multiple mRNA targets encoding virulence factors (*spa*, *coa*. SA1000, *rot*), indirectly activates levels of secreted proteins (Efb)/Δ*rnc* strain was attenuated in a mouse infection model	*S. aureus*	[44, 71, 72, 97]
RNase Y (*rny*)	endoribonucleolytic	Repression		Affects expression of virulence factors through* agr* or independently/affects virulence in silkworm and mouse models	*S. aureus*	[81, 83]
RNase Y	Endoribonucleolytic	Repression		Affects expression of several virulence factors/affects virulence in silkworm and mouse models	*S. pyogenes *	[81, 82]
RNases J1/J2 (*rnjA/rnjB*)	Endoribonucleolytic, 5′-3′ exoribonucleolytic	Repression		Affect bulk mRNA decay and expression of multiple virulence factors	*S. pyogenes*	[78–80]
RNase J2	Endoribonucleolytic, 5′-3′ exoribonucleolytic	Activation		*ebp* operon (pilus proteins)/*rnjB* is essential for biofilm formation	*E. faecalis*	[68]
Unknown		Activation	FasX	Stabilizes *ska* mRNA encoding streptokinase	*S. pyogenes*	[70]
Unknown		Activation	VR-RNA	Activates translation of *colA* mRNA encoding toxin collagenase	*C. perfringens*	[90]
Unknown		Repression	LhrA and Hfq	Represses the synthesis of ChiA protein (chitinase)	*L. monocytogenes*	[100, 101]
Csn1/ RNase III	Endoribonucleolytic/dsRNA-specific endoribonucleolytic	Repression	tracrRNA	Targets foreign invading DNA such as plasmid and phages	*S. pyogenes, Listeria innocua, Streptococcus mutans, Streptococcus thermophilus *	[102]
Catalytic ribozyme	Alternative splicing	Activation	Riboswitch with bound c-di-GMP	Activates the translation of a putative virulence gene	*C. difficile*	[109, 110]
PemK	Endoribonucleolytic	Repression		Affects global RNA decay	*B*.* anthracis *	[124]
MazF, Txe	Endoribonucleolytic	Repression		Localizes with vancomycin resistance genes, confers plasmid stabilization	*enterococci*	[125, 126]
MazF	Endoribonucleolytic	Repression		Cleaves several mRNAs (*sraP, spa* and *hla*) encoding virulence factors, and possibly other mRNAs	*S. aureus*	[128–131, 133]
MazF, VapC	Endoribonucleolytic	Repression		Several TA modules are upregulated during hypoxia and infection of macrophages	*M. tuberculosis*	[113, 135, 136]
