# Supplementary material for: When Ribonucleases Come into Play in Pathogens: A Survey of Gram-Positive Bacteria
Source: Int J Microbiol. 2012 Mar 13;2012:592196. doi: 10.1155/2012/592196 (PMC3328962; doi:10.1155/2012/592196)
Supplement: Supplementary file 1 — In the supplementary material, the genomic context for the genes encoding the ribonucleases rnpA, rnc, rnjA/rnjB, pnpA, rny as well as the pyrophosphohydrolase rppH and the helicase cshA is shown for several major Gram-positive pathogenic bacteria, namely, Listeria monocytogenes, Staphylococcus aureus, Mycobacterium tuberculosis, Enterococcus faecalis, Clostridium difficile, and Bacillus anthracis. [file 592196.f1.pdf]

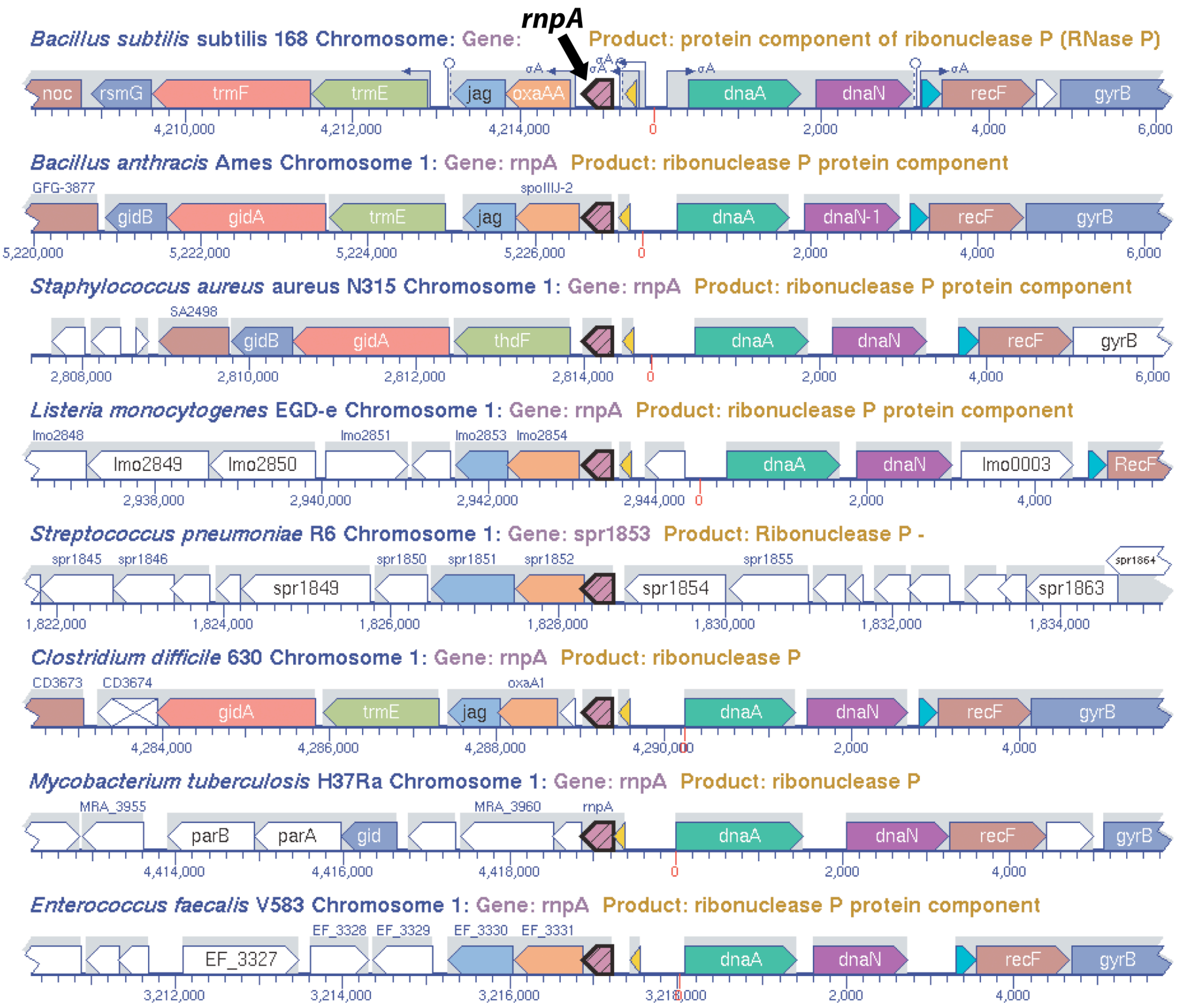

Supplementary Figure 1. Alignment of the genomic context for RNase P (protein subunit) (*rnpA*). Alignment made using www.Biocyc.org.

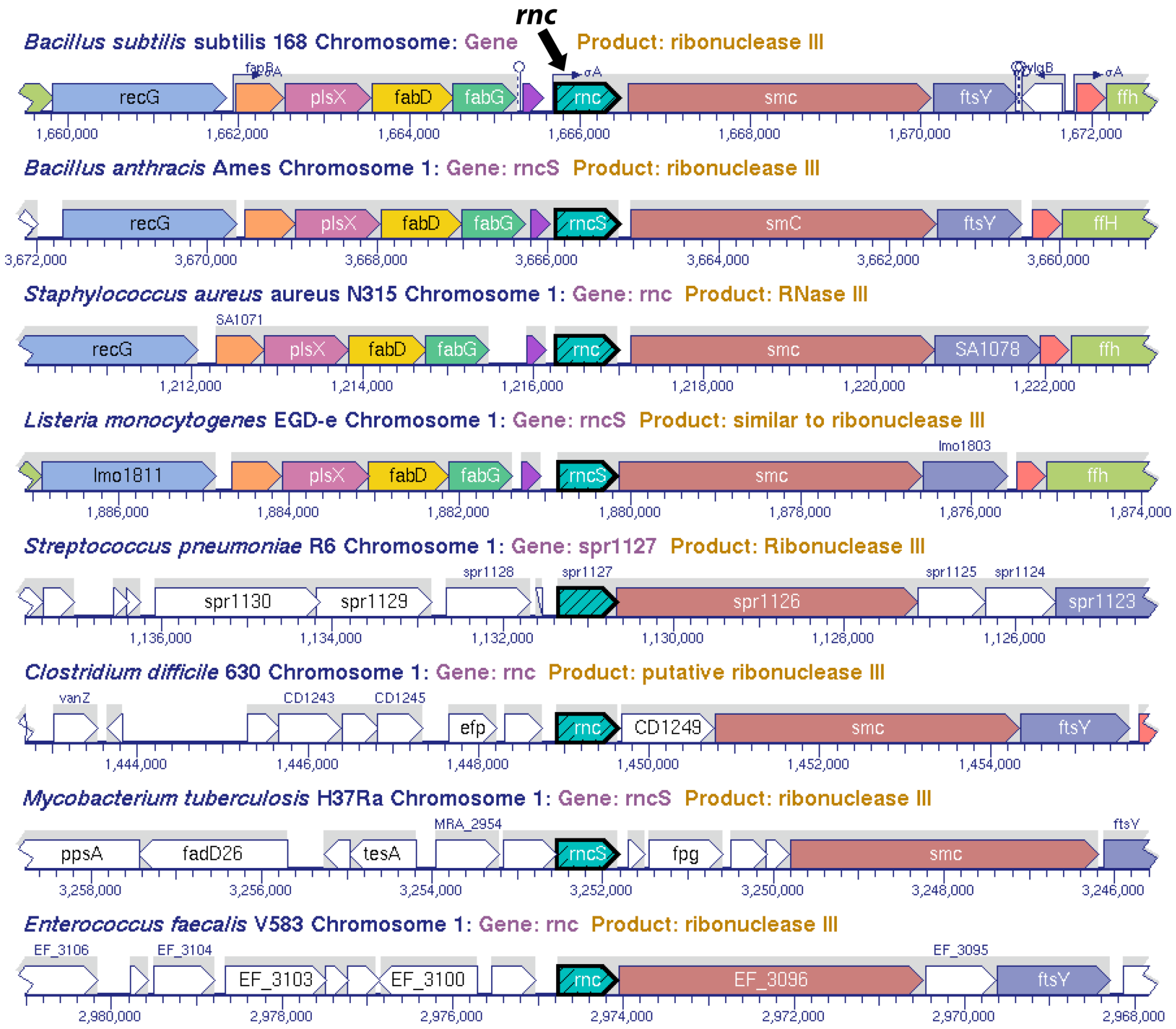

Supplementary Figure 2. Alignment of the genomic context for RNase III (*rnc*). Alignment made using www.Biocyc.org.

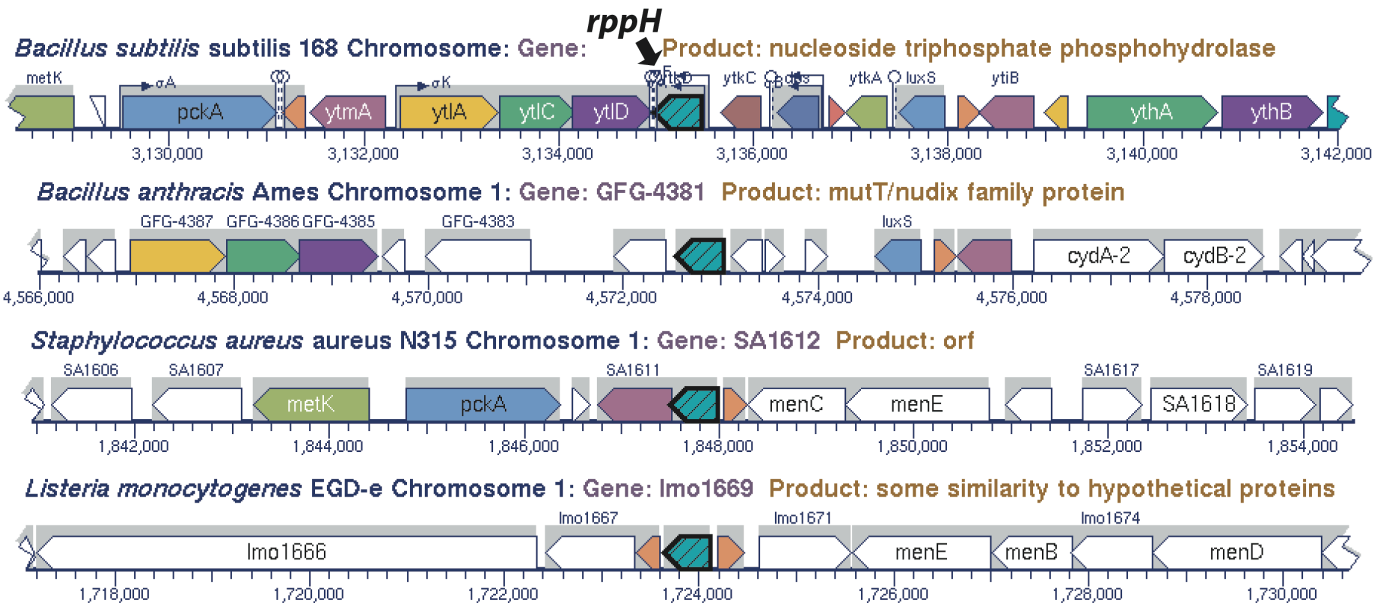

Supplementary Figure 3. Alignment of the genomic context for RppH (*rppH*). Alignment made using www.Biocyc.org.

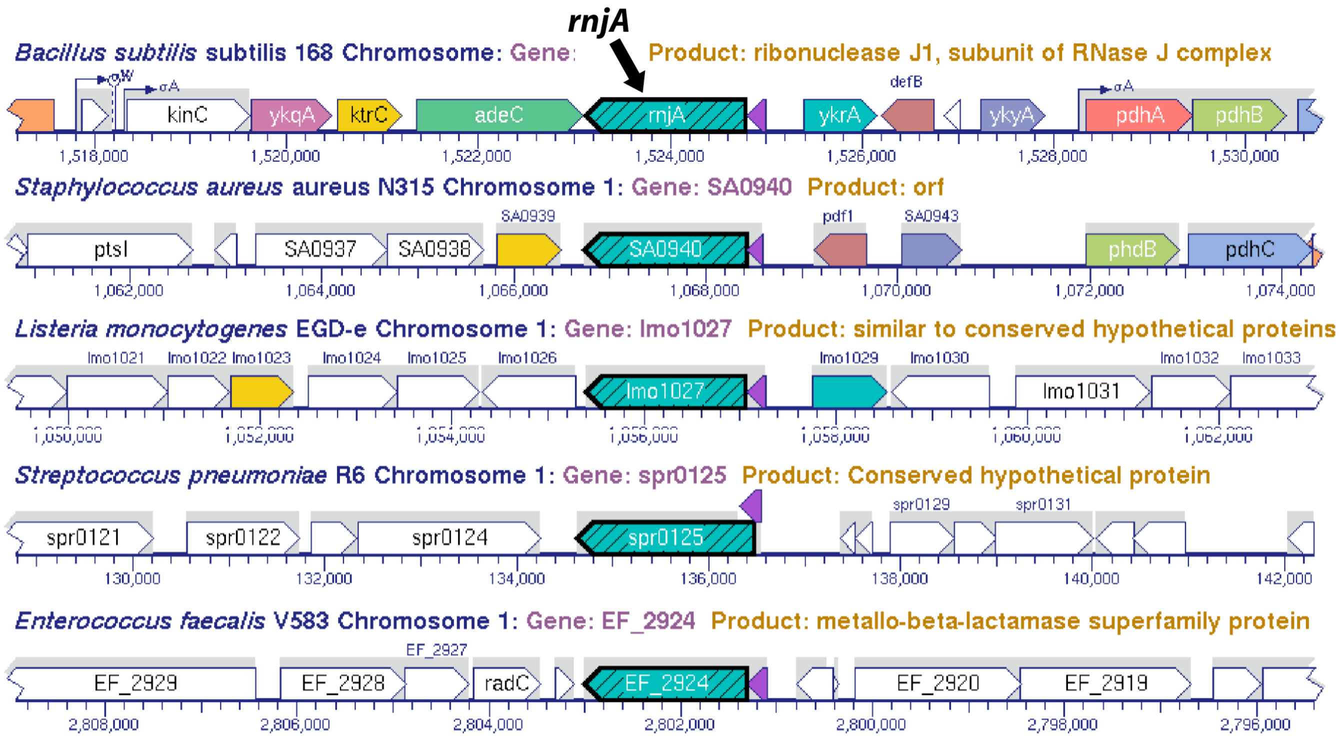

Supplementary Figure 4. Alignment of the genomic context for RNase J1 (*rnjA*). Alignment made using www.Biocyc.org.

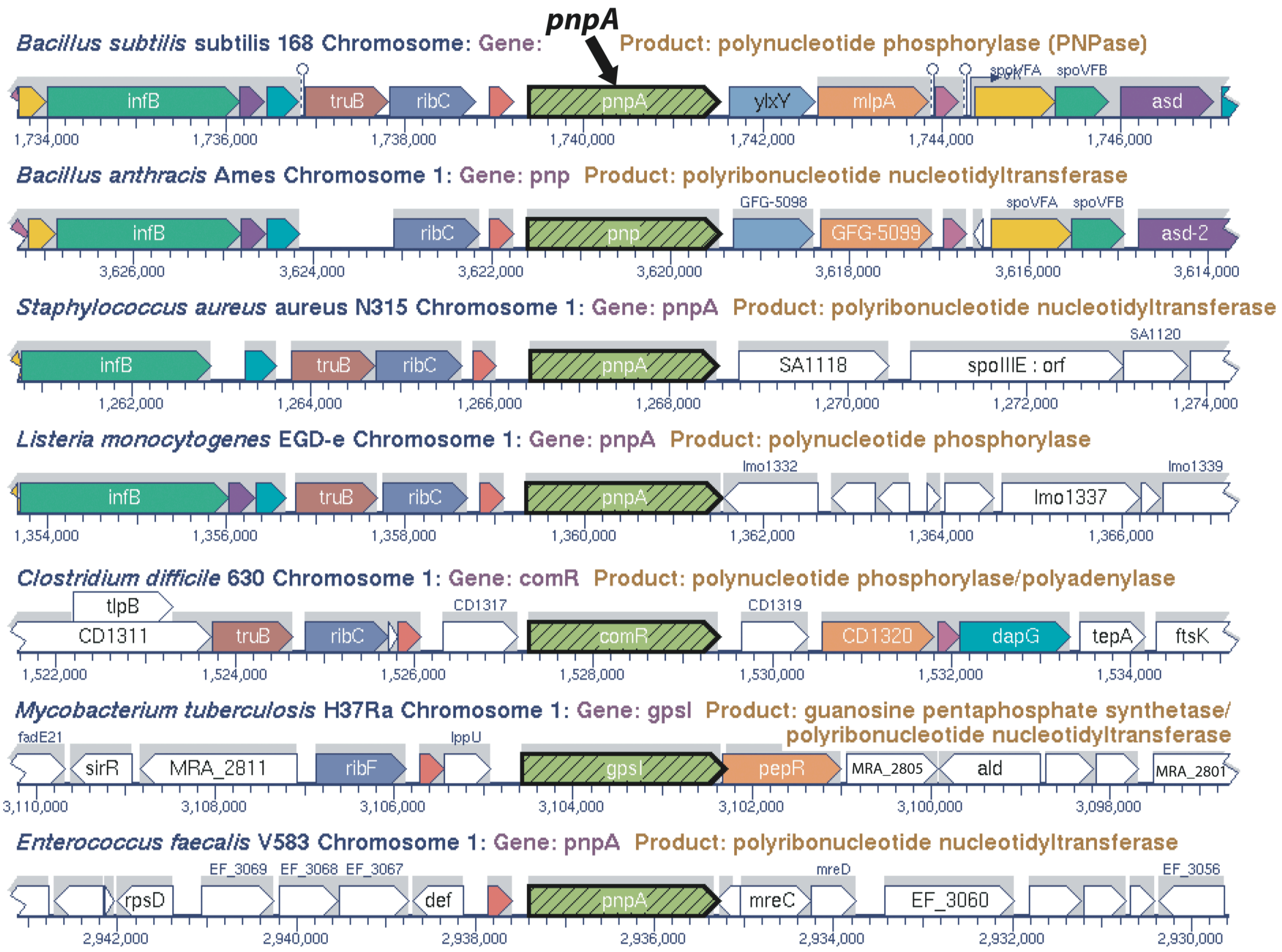

Supplementary Figure 5. Alignment of the genomic context for PNPase (*pnpA*). Alignment made using www.Biocyc.org.

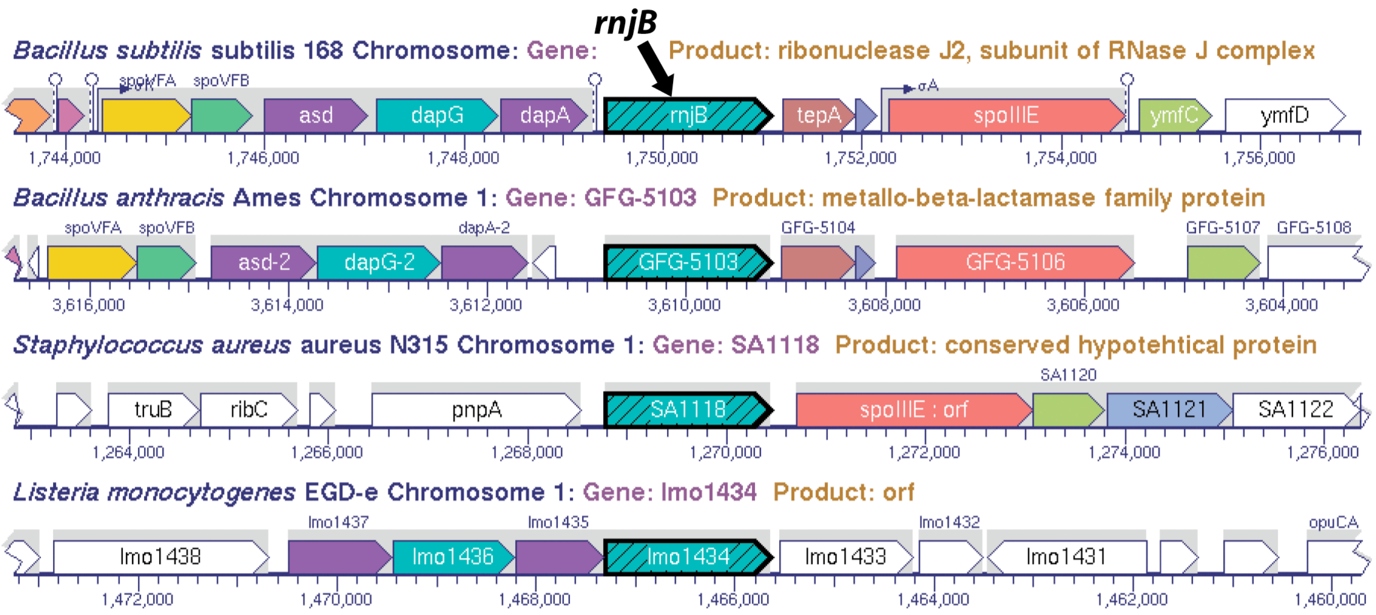

Supplementary Figure 6. Alignment of the genomic context for RNase J2 (*rnjB*). Alignment made using www.Biocyc.org.

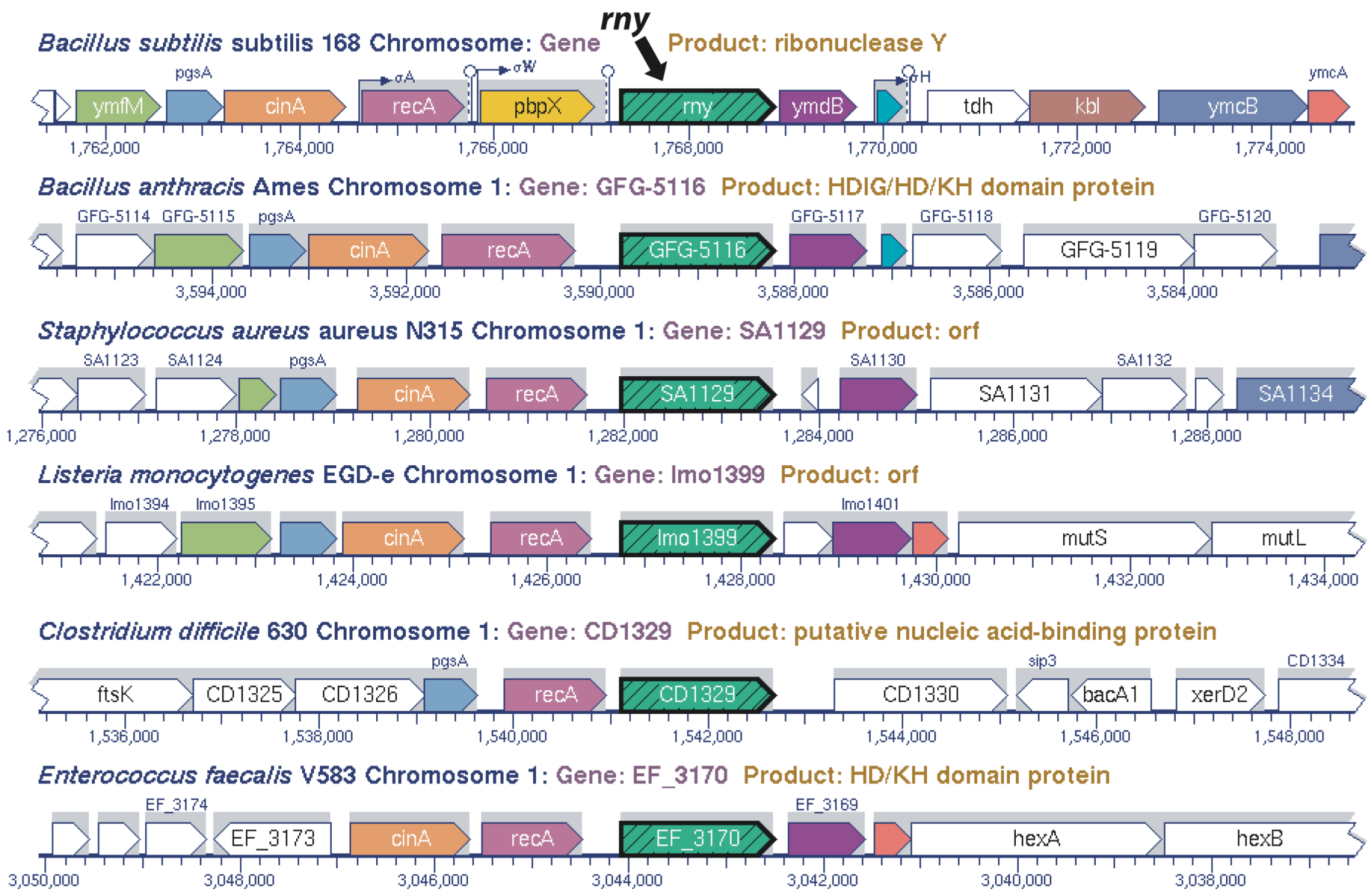

Supplementary Figure 7. Alignment of the genomic context for RNase Y (*rny*, previously *ymdA*). Alignment made using www.Biocyc.org.

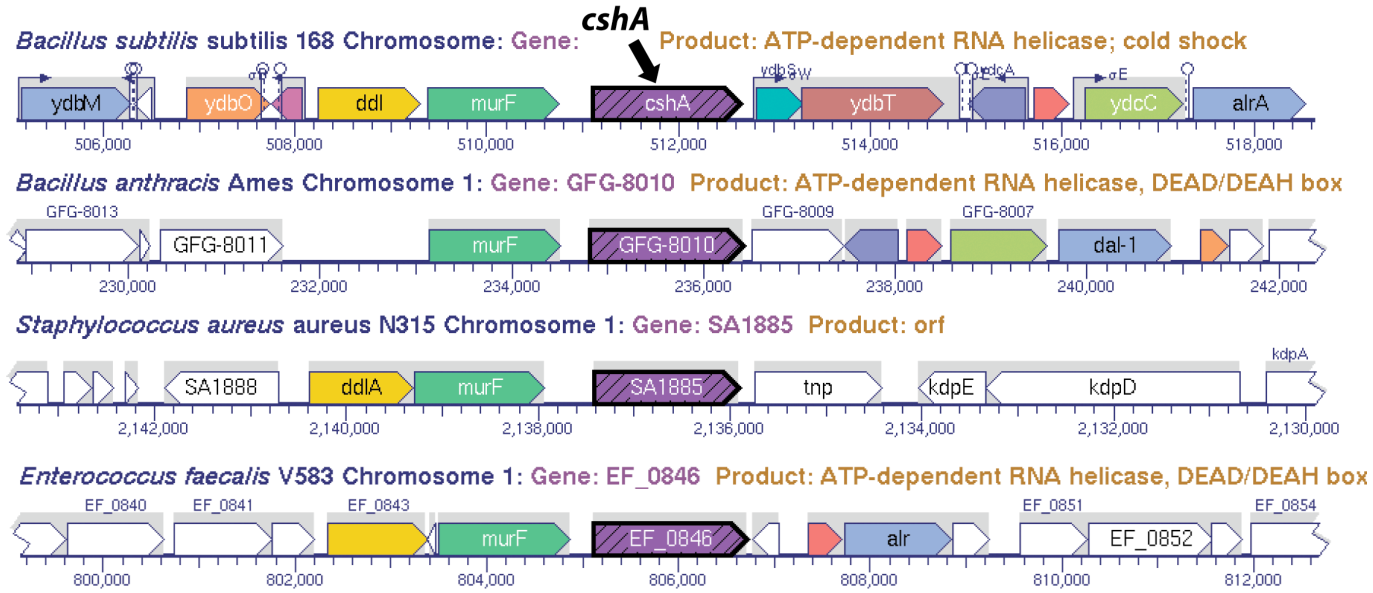

Supplementary Figure 8. Alignment of the genomic context for CshA (*cshA*). Alignment made using www.Biocyc.org.
